# The Utility of Positron Emission Tomography in the Treatment Planning of Image-Guided Radiotherapy for Non-Small Cell Lung Cancer

**DOI:** 10.3389/fonc.2014.00273

**Published:** 2014-10-07

**Authors:** Alexander Chi, Nam P. Nguyen

**Affiliations:** ^1^Department of Radiation Oncology, Mary Babb Randolph Cancer Center, West Virginia University, Morgantown, WV, USA; ^2^International Geriatric Radiotherapy Group, Tucson, AZ, USA

**Keywords:** NSCLC, IGRT, PET, target volume delineation, treatment planning

## Abstract

In the thorax, the extent of tumor may be more accurately defined with the addition of ^18^F-fluorodeoxyglucose (FDG) positron emission tomography (PET) to computed tomography (CT). This led to the increased utility of FDG-PET or PET/CT in the treatment planning of radiotherapy for non-small cell lung cancer (NSCLC). The inclusion of FDG-PET information in target volume delineation not only improves tumor localization but also decreases the amount of normal tissue included in the planning target volume (PTV) in selected patients. Therefore, it has a critical role in image-guided radiotherapy (IGRT) for NSCLC. In this review, the impact of FDG-PET on target volume delineation in radiotherapy for NSCLC, which may increase the possibility of safe dose escalation with IGRT, the commonly used methods for tumor target volume delineation FDG-PET for NSCLC, and its impact on clinical outcome will be discussed.

## Introduction

In recent years, ^18^F-fluorodeoxyglucose (FDG) positron emission tomography (PET) has emerged to be an essential tool in the staging of non-small cell lung cancer (NSCLC) (1). Tumor imaging through FDG-PET is achieved based on the difference in glucose metabolism between malignant and normal tissue, which leads to relatively increased FDG accumulation in tumor cells. FDG undergoes positron emission decay, which ultimately leads to the production of a pair of positron annihilation gamma (γ) rays (511 keV each) traveling in opposite directions (2). These two gamma rays are then detected by two opposing coincidence detectors in a PET scanner for imaging (2). Because of the ability of FDG-PET to detect malignancy prior to the development of any noticeable anatomical changes, it was consistently found to have superior sensitivity and specificity in the staging of lung cancer (3, 4). This is especially true for mediastinal staging. As shown in a meta-analysis by Gould et al., FDG-PET has superior median sensitivity and specificity over CT (85 vs. 61%, 90 vs. 79%, *p* < 0.001) in the identification of lymph node involvement by NSCLC (5). CT’s median specificity improves to be superior to FDG-PET in the evaluation of enlarged lymph nodes in the same study (93 vs. 78%, *p* = 0.002). However, FDG-PET may provide additional information on the extent of tumor involvement at the primary site and in the regional lymph nodes during target volume delineation for radiotherapy planning in the treatment of NSCLC to avoid geometric tumor miss, and unnecessary inclusion of normal tissue. In the following sections, the impact of FDG-PET on radiotherapy target volume delineation for NSCLC, which may increase the likelihood of dose escalation with IGRT, the commonly used methods of defining gross tumor on FDG-PET, 4D-PET/CT imaging, and FDG-PET’s impact on treatment outcome will be discussed.

## Impact of FDG-PET on Target Volume Delineation

**Figure 1:**
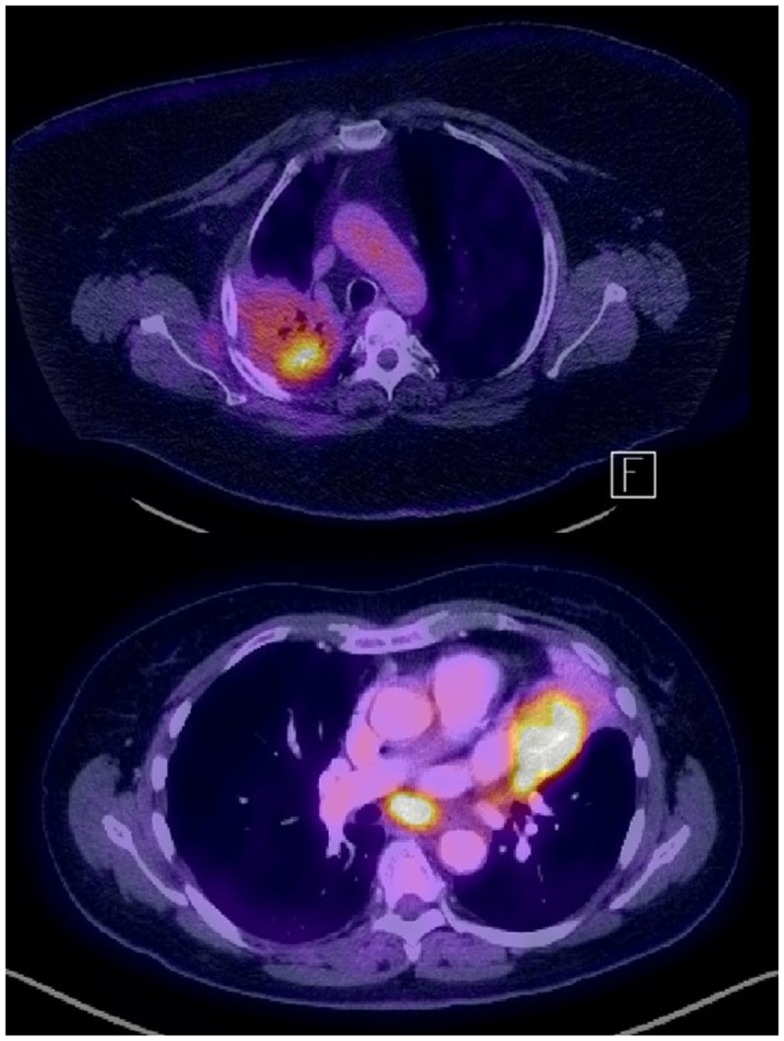
**Examples of PET-avid NSCLC in the presence of fibrosis (recurrence after chemo-radiation, top) and atelectasis (bottom)**.

**Figure 2:**
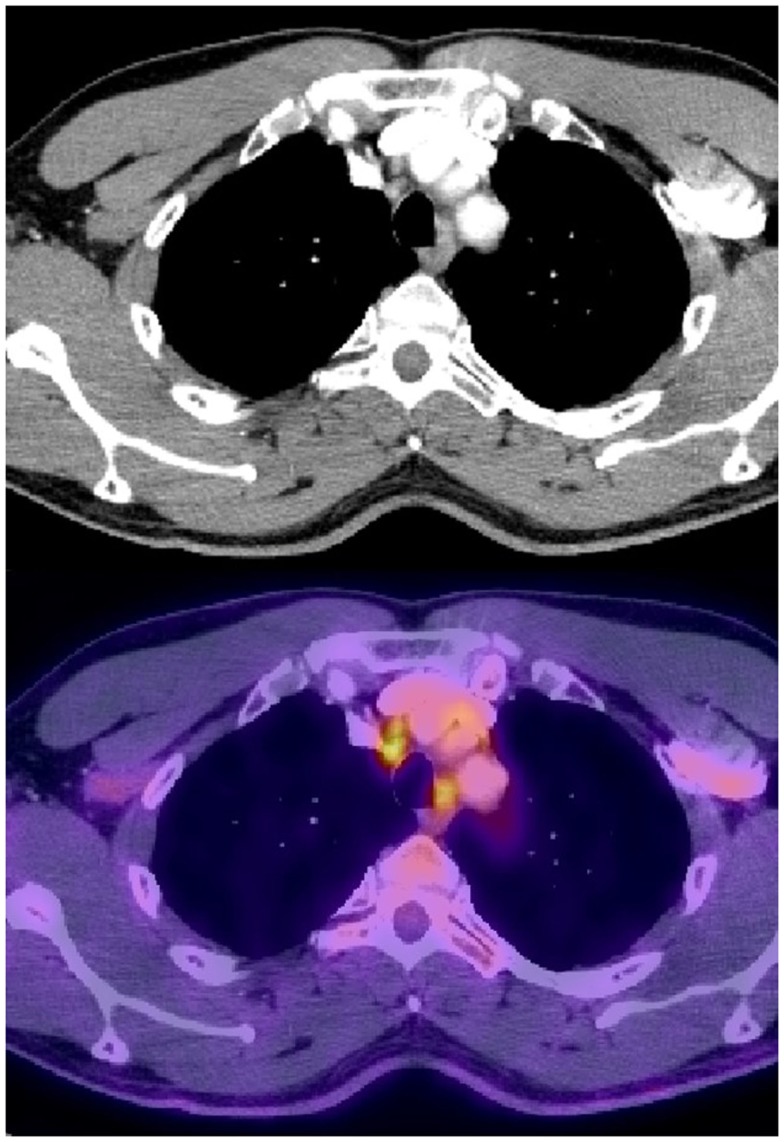
**Normal sized mediastinal lymph nodes (2R) that were PET avid and were biopsied positive in a patient with stage IIIB adenocarcinoma of the right lower lobe**.

The incorporation of FDG-PET during target volume delineation has frequently led to changes in the shape and size of the target volumes; as well as the tumor stage when FDG-PET was not done as a part of the initially staging evaluation in patients with NSCLC. This fact has been well illustrated in multiple studies (6–14). As shown in Table 1, changes in the target volumes of over 20% and stage alteration of 20–50% have been consistently observed when FDG-PET was incorporated in target volume delineation and when FDG-PET was not a part of the initial staging studies. Most prominent changes are often associated with the presence of atelectasis in the treated areas (Figure 1), or the identification of additional nodal disease, which is difficult to visualize on CT (6–9, 11, 14) (Figure 2). This is well illustrated by Bradley et al., who demonstrated PTV and stage alteration of 58 and 31% in patients with stage I-III NSCLC when FDG-PET was incorporated in target volume delineation (9). Among 24 patients planned for definitive three-dimensional conformal radiotherapy (3D-CRT), PET led to a GTV reduction in 3 patients with atelectasis, and an increase in GTV due to the identification of additional regional nodal disease in 10 patients, and the identification of an additional parenchymal disease in 1 patient. GTV-reduction due to the utilization of PET resulted in dose reduction to the normal lungs and esophagus in patients with tumor-related atelectasis in this study, which suggests a potential advantage in the sparing of thoracic organs at risk (OAR) with the incorporation of FDG-PET in target volume delineation. This is corroborated in similar studies, which demonstrated similar PET-related target volume alterations, and the resulting decrease in the dose to the heart, esophagus, spinal cord, and the normal lungs (7, 8, 11, 12, 14). In one study, PET-related exclusion of metabolically inactive lymph node and atelectasis resulted in GTV reduction of 39 and 84%, respectively, which led to the reduction of the mean lung dose (MLD) and volume of the normal lungs receiving 20 Gy (V_20_) by 6.1 Gy and 12% on average (11). In the same study, the median dose to the spinal cord was reduced from 45.7 to 41.7 Gy with the incorporation of FDG-PET in target volume delineation (*p* < 0.05). In another study, GTV reduction was observed in 73.3% of patients with stage III NSCLC in the presence of atelectasis, which possibly led to statistically significant decrease in commonly used dosimetric parameters, such as V_20_ for the normal lungs, and V_55_ for the esophagus (14).

**Table 1:** **FDG-PET-related alteration of target volumes in NSCLC**.

Reference	Stage	Volume changes due to FDG-PET	Dosimetric impact
Nestle et al. (6)	IIIB-IV	Change in size and shape of radiation fields: 35%	
		Field size reduction: 26% (median 19.3%)	
		More changes observed in the presence of atelectasis (*p* = 0.03)	
Erdi et al. (7)	Unknown	PTV increase (additional nodal disease): 19%^a^	Mean heart dose decreased by 50% in the PET plan in one case
		PTV reduction: 18%^a^	
Mah et al. (8)	III (2/7)	Stage alteration: 23%	Maximum spinal cord dose is decreased on average with PET/CT-based planning (*p* ≤ 0.01)
		PTV reduction and increase among three observers: 24–70 and 30–76%	
Bradley et al. (9)	I–III (65% stage III)	Stage alteration: 31%	Alteration of the GTV led to corresponding changes in the dose to the esophagus and the normal lungs
		PTV alteration: 58%	
		GTV reduction (atelectasis): 12%	
		GTV increase (additional primary and nodal disease): 46%	
van Der Wel et al. (10)	III	Nodal GTV decreased by 3.8 cm^3^ on average (*p* = 0.011) Radiation field change: 66.7% (decreased in 52.4%, increased in 14.3%)	Alteration of the GTV led to corresponding changes in dose to the esophagus and the normal lungs
			PET enabled dose escalation from 56 Gy to 71 Gy on average (*p* = 0.038) & increased TCP by at least 6% on average (*p* < 0.05)
Ceresoli et al. (11)	66.7% III	Stage alteration: 48% ≥25% change in GTV: 39%	Dose reduction to the spinal cord was observed in PET plans (median 41.7 Gy vs. 45.7 Gy, *p* < 0.05)
			Changes in GTV led to corresponding changes in dose to normal lung tissue
		5/7 with GTV increase (additional nodal disease)	
		2/7 with GTV reduction (PET negative enlarged LN and atelectasis)	
Faria et al. (13)		Stage alteration: 44%	
		GTV alteration: 56%	
		Decrease: 37.3%	
		Increase: 18.7%	
Yin et al. (14)	III^b^	GTV alteration: 100% (≥25 in 40% of patients)	PET led to significant changes in V_20_, V_30_ for the lungs and V_50_, V_55_ of the esophagus
		Decrease: 73.3% (155.1–111.4 cm^3c^)	
		Increase: 26.7% (125.8–144.7 cm^3c^)	

*^a^Average; TCP, tumor control probability*.

*^b^Atelectasis present in all patients*.

*^c^Median*.

PET-related increase in the GTV has been mainly due to the identification of additional regional nodal disease (Table 1). This has been shown to result in an increase in the dose to the surrounding normal tissue (9, 11). However, this increase may not be clinically significant in all patients. As shown by Ceresoli et al., PET-related increase in GTV only resulted in an increase of the MLD by 1.08 Gy, and the V_20_ by 2.4% on average (11). In addition, incorporation of FDG-PET in the delineation of regional nodal disease may lead to a decrease in the nodal GTV. This has been demonstrated in patients with N2-N3 disease by van Der Wel et al., who showed a PET-related decrease of the nodal GTV from 13.7 ± 3.8 to 9.9 ± 4.0 cm^3^ (*p* = 0.011) (10). It led to significant decrease in radiation dose to the esophagus (V_55_ decreased from 30.6 ± 3.2 to 21.9 ± 3.8%, *p* = 0.004); and the normal lungs (V_20_ decreased from 24.9 ± 2.3 to 22.3 ± 2.2%, *p* = 0.012). As a result, dose escalation from 56.0 ± 5.4 to 71.0 ± 13.7 Gy (*p* = 0.038) became feasible, which led to improved TCP from 14.2 ± 5.6 to 22.8 ± 7.1% (*p* = 0.026) without accounting for geometric misses, and improved TCP from 12.5 to 18.3% when that is accounted for (*p* = 0.009). These findings further demonstrate the advantage of incorporating FDG-PET information in target volume delineation especially for stage III NSCLC, which makes dose escalation possible.

To further investigate the accuracy of FDG-PET in identifying nodal disease, 73 NSCLC patients with known positive lymph nodes by CT, or PET and pathology data for all suspected lymph nodes were further assessed by Vanuytsel et al. (12). Using PET-CT data, inclusion of pathological nodes in the nodal GTV was found to increase from 75% with CT alone to 89% (*p* = 0.005). In their study, PET-related GTV alteration was observed in 62% of the patients. Among them, PET-related GTV increase was observed in 16/45 patients. While 11 of these 16 patients’ GTV increase was supported by pathologic findings, it was unnecessary in five patients. PET incorporation resulted in GTV reduction in 29/45 patients. Twenty-five of them were correlated with pathological findings. Overall, 80% of all the PET-related GTV alterations were correct and inappropriate changes often were due to low tumor burden that is beyond the resolution of FDG-PET, or misinterpretation of the location of nodal disease. Pathology correlation in this study supports the utilization of FDG-PET in the delineation of nodal disease for NSCLC, which is shown to be more accurate than CT alone. The improved accuracy in identifying nodal disease with FDG-PET was shown by Faria et al. as well (13). However, how to improve the accuracy of PET-based identification of nodal disease from NSCLC remains to be investigated in the future. PTV reduction due to PET-related GTV reduction was again demonstrated in the study by Vanuytsel et al. in 10 selected stage III NSCLC patients, which led to a decrease of V_20_ of the normal lungs by 27 ± 18% (*p* = 0.001) (12). Thus, further demonstrates an advantage in OAR sparing with incorporation of PET information in target volume delineation for NSCLC, which may increase the likelihood of dose escalation in the treatment of loco-regionally confined NSCLC with definitive radiotherapy.

## Methods of Target Volume Delineation on FDG-PET

Given the multiple variables that exist in PET imaging for NSCLC (2, 3), there is no consensus on how to best delineate gross tumor on FDG-PET at the current time. Visual interpretation of the PET or PET/CT images with an expert nuclear medicine physician remains to be a frequently used approach when delineating the GTV. The maximum standardized uptake value (SUV_max_) was quantitatively used to determine FDG-PET activity because it is the most consistent and reliable parameter used to assess tumor activity in clinical practice. It is defined as the maximum tumor concentration of FDG divided by the injected dose of FDG, corrected for the body weight of the patient [SUV_max_ = maximum activity concentration/(injected dose/body weight)]. In 87 patients with malignant and benign focal pulmonary lesions who had a firm pathological diagnosis and at least 2 years of follow up, the sensitivity, specificity, and accuracy of 97, 82, and 92% were found when a SUV threshold of 2.5 was used for the diagnosis of lung cancer (15). This SUV threshold of 2.5 was proposed to be used as a cut-off for GTV delineation in radiotherapy planning (16). Slightly lower SUV threshold of 2 ± 0.4 has been proposed based on the PET/CT of 19 patients with stage II-III NSCLC, which could be distinctively visualized (17). Alternatively, fixed threshold from 36 to 44% of the SUV_max_ based on the source-to-background ratio for volumes larger than 4 mL has been shown to accurately identify the tumor volume in phantoms (18).

Various approaches of PET-GTV delineation of the primary tumor were compared in a study by Nestle et al. (19). The fixed 40% thresholding method was found to be inadequate especially in the setting of inhomogeneous FDG-uptake within the tumor. However, PET-GTV contoured based on direct visualization, the SUV ≥2.5, and an algorithm accounting for the source-to-background FDG-uptake ratio all correlated well with GTV of the primary tumor contoured on CT. The poor correlation between CT-based GTV and PET-GTV generated with percent thresholding was also demonstrated in a study by Devic et al. (20). Upon further analysis of 20 peripheral NSCLC, the optimal threshold was found to be dependent on tumor size: 15 ± 6% for tumors >5 cm, 24 ± 9% for tumors 3–5 cm, 42 ± 2% for tumors <3 cm (21). Larger SUV_max_ was found in larger tumors in this study. Thus, a single fixed percent-threshold method of GTV delineation appears to be inadequate and this may be due to multiple factors, such as the background FDG-uptake, heterogeneous FDG-uptake in the tumor, as well as respiratory motion and tumor size.

Multiple studies have attempted to investigate how well different GTV delineation strategies correlate with the true tumor volume in surgical specimens for NSCLC (Table 2). In correlation with surgical pathology findings, PET/CT has been shown to be more accurate than CT or FDG-PET alone in the estimation of tumor size for NSCLC (22, 23). In a study of 37 patients, the mean SUV at the edge of the PET tumor halo which corresponded to the edge of the tumor on pathology was 2.41 ± 0.73 (22). In a different study, GTV delineated on PET/CT using a SUV cut-off value of 2.5 resulted in the best correlation with the pathological tumor volume (23). In an analysis of 15 lobectomy specimens after PET/CT imaging, the most optimal percent threshold, and absolute SUV cut-off that correlated with the pathologic tumor volume (GTV_path_) were found to be 31 ± 11%, and 3.0 ± 1.6, respectively (24). Only the SUV percent threshold was correlated with the GTV_path_ and the tumor diameter in this study (*p* < 0.05). However, limitations have been observed with both approaches of GTV delineation based on pathological correlation. The SUV cut-off at the edge of the tumor on PET has been shown to be dependent on tumor size and histology by Lin et al. (22). In their study, higher mean SUV is observed with tumors over 3 cm and of squamous histology. In contrary to the studies described above, thresholding has been shown to be less accurate than CT in predicting the maximal tumor dimension in pathological tumor specimens in 31 patients who underwent lobectomy shortly after PET/CT (25). The uncertainties associated with percent thresholding or the use of an absolute SUV cut-off for GTV delineation appear to be influenced by the background FDG concentration and the tumor size, which are reflected by the mean SUV. To minimize the impact of these factors, it was proposed to adjust percent thresholding based on the mean target SUV in order to accurately define the gross tumor (26).

**Table 2:** **Methods of GTV delineation on PET in correlation with surgical specimens**.

	Patient no.	Method of GTV delineation on PET	Correlation between CT, PET, PET/CT, and pathological tumor size
Lin et al. (22)	37	Halo for tumor observed in fused PET-CT images	Stronger correlation between GTV and pathological tumor dimensions were observed with PET/CT
			Mean SUV of the external margin of halo was 2.41 ± 0.73
			T stage and histology significantly influenced SUV at the edge of the halo
Yu et al. (23)	52	SUV of 2.5	FDG-PET/CT has significantly better correlation with surgical specimens than CT or PET alone, especially in the presence of atelectasis
Yu et al. (24)	15		Best correlation between PET GTV and the actual tumor was found at the SUV threshold of 31 ± 11%, and absolute SUV cut-off of 3.0 ± 1.6
Wu et al. (25)	31	Thresholding with 20–55% of SUV_max_	Maximal primary tumor dimension was more accurately predicted by CT at the window-level of 1,600 and −300 HU than PET GTVs (best correlation with pathological tumor volume at 50% SUV_max_)
Schaefer et al. (27)	15	Tumor threshold = A*mean SUV_70%_ + B*background	Pathological tumor volume: 39 ± 51 mL
			PET tumor volume: 48 ± 62 mL
			CT tumor volume: 60.6 ± 86.3 mL
			Both CT and PET volumes are highly correlated with pathological volumes (*p* < 0.001).
			Increased variation between PET and pathological tumor volumes were observed in lower lobes
van Baardwijk et al. (28)	33	Source-to-background ratio auto-segmentation	Maximal tumor diameter of the PET GTV is highly correlated with that in surgical specimens (CC = 0.90). Auto-segmented GTVs are smaller than manually contoured GTVs on PET/CT
Wanet et al. (31)	10	Gradient-based method	Comparison of both CT and PET GTV
		Fixed threshold at 40 and 50% of the SUV_max_.	Gradient-based method led to the best estimation of the GTV
		Adaptive thresholding based on the source-to-background ratio	PET GTVs were smaller than CT GTVs in general
Cheebsumon et al. (32)	19	Absolute SUV cut-off (2.5)	Adaptive 50% and gradient-based methods generated the most consistent maximal tumor dimension, which had a fair correlation with the pathological tumor size
		Fixed threshold at 50% and 70% SUV_max_	
		Adaptive thresholding 41–70% SUV_max_	
		Contrast-oriented algorithm	
		Source-to-background ratio	
		Gradient-based method	

To account for the effects of tumor volume and background FDG concentration, a contrast-oriented thresholding algorithm (COA) was proposed for the delineation of PET GTV for NSCLC (27). This approach was shown to reduce the GTV volume when compared to CT alone. Also, it was shown to be highly correlated to the pathological tumor volume. Similar findings were obtained in a study of 33 patients with NSCLC when a source-to-background ratio based auto-segmentation approach was used (28). These studies demonstrate the feasibility of an adaptive thresholding approach for GTV delineation on PET. However, higher variation between pathological and PET tumor volumes were observed in the lower lobes with the COA, suggesting respiratory motion to be a source of inaccuracy in GTV delineation on PET (27).

A gradient-based approach for PET-GTV delineation has been proposed to minimize the statistical noise, and resolution blur (more pronounced in the setting of large respiration induced tumor motion) (29). When compared to other methods of GTV delineation on PET, this method was found to be the most accurate in a phantom study by Werner-Wasik et al. (30). This approach was also compared with other methods of GTV delineation in surgical specimen correlations studies (31, 32). It was found to be superior to manual, fixed thresholding at 40 and 50%, and the source-to-background ratio methods of PET-GTV delineation, and manual CT GTV delineation on 4D-PET/CT in 10 patients with stage I-II NSCLC who underwent lobectomy (31). In another study of 19 patients who underwent free-breathing PET/CT prior to surgery, the gradient method was found to be highly correlated with the maximal tumor size in surgical specimens as well (32). Thus, the gradient-based method is highly promising, which warrants further investigation in future trials. While the various methods discussed are shown to be feasible, they are often confounded by factors, such as statistical noise, blurring effect due to respiratory motion, and uncertainties in the estimation of pathological tumor size in surgical correlative studies. Thus, further studies need to be conducted to explore what would be the best method for the most accurate GTV delineation on PET.

## Improving PET-GTV Delineation with 4D-PET/CT

Respiratory motion often causes blurring and alteration of the FDG-uptake within the tumor, which lead to uncertainties in the delineation of the gross tumor volume on PET (33). These uncertainties may potentially be minimized with 4D-PET/CT imaging for more accurate identification of the true extent of the tumor in various portions of the respiratory cycle, and low volume disease, which may be missed on free-breathing PET/CT (34, 35). As shown by Lamb et al., tumor volumes delineated on 4D-PET not only correlates better with that delineated on 4D CT, but also enhances the estimation of the true extent of tumor in the vicinity of similar density soft tissues, such as the diaphragm, chest wall, and the heart (36). Thus, the GTV delineation on PET can be improved with 4D-PET/CT imaging. This is, especially, helpful in image-guided radiotherapy (IGRT) due to the very small PTV margins used, which allows for dose escalation to the gross disease without significantly increase the risk of severe toxicities to normal thoracic structures. Therefore, 4D-PET-based tumor target delineation should be used as often as possible when a high dose of radiation is delivered in the thorax.

## Delineation of Nodal Disease on PET

The delineation of regional nodal disease on PET has been conducted in similar ways as that for the primary tumor. Various methods were compared by Nestle et al., who again demonstrated that an algorithm accounting for the source-to-background FDG-uptake ratio was superior to direct visualization, 40% thresholding, or the SUV ≥2.5 cut-off methods (37). Furthermore, the nodal volume delineated on PET tends to be larger than that delineated on CT, which was felt to be possibly caused by respiratory motion. This was corroborated in a study on 4D-PET-based nodal disease delineation (38). As shown in this study, a 3D nodal internal target volume (ITV) expansion of over 1 cm is required to cover 91% of the lymph nodes while accounting for respiratory motion. While it is still inadequate in situations of highly mobile lymph nodes. On the contrary, 4D-PET-based ITV was able to not only adequately encompass nodal disease in the setting of respiratory motion, but also sparing additional normal tissue (45 ± 34 cm^3^) when compared with 3D nodal ITV generated with large margins that would be required to account for respiratory motion in the majority of the cases. Thus, 4D-PET imaging may improve precise and accurate localization of mediastinal disease over CT, which can potentially improve targeting in the mediastinum for the delivery of IGRT in the treatment of lung cancer.

## Clinical Outcome Following PET-Based Planning

In recent years, two studies have reported the clinical outcome following concurrent chemo-radiation for stage II-III NSCLC when the target volumes were delineated based on FDG-PET findings (39, 40). In a pilot study of 32 patients, only one regional failure and one local progression were observed shortly after concurrent chemo-radiation when only PET-avid disease was included in the target volume (39). The nodal failure was later identified to be a missed PET-avid lymph node that was not included in the target volume. In another study of 137 patients with stage III NSCLC, local-regional recurrence alone as the first event was only 14.6%, while that combined with distant metastasis as the first event was 16.8% following concurrent chemo-radiation to a median dose of 65 ± 6 Gy when only PET-avid disease was treated (40). These findings suggest that PET-based planning may lead to at least equivalent clinical outcomes when compared with CT-based planning (41). However, additional normal tissue sparing may be achieved with PET-based GTV delineation, which may aid dose escalation to the primary tumor to improve the local control of locally advanced NSCLC. As suggested in a meta-analysis, this may potentially improve patient survival (42).

## Novel PET Tracers for Dose Painting

Residual disease at the primary tumor site can often be identified on the pre-radiotherapy PET, which may be treated with a higher dose with dose painting through IMRT to enhance local control of the primary tumor (43). To better identify radio-resistant tumor cells within the primary tumor, hypoxia imaging with PET has been explored in recent years. PET with hypoxia tracers, such as F-MISO, 18F-FAZA, or 18F-HX4, have been shown to be able to identify areas of hypoxia in multiple cancers, including lung cancer (44–46). This may help identify areas at a higher risk for tumor recurrence, which may need to be treated with a higher daily dose than the remaining portions of the gross tumor with dose painting (47, 48). As of current, dose painting to deliver a higher dose to areas of higher radio-resistance remains to be further investigated.

## Conflict of Interest Statement

The Guest Associate Editor Ulf Lennart Karlsson declares that, despite having collaborated with Nam P. Nguyen, the review process was handled objectively and no conflict of interest exists. The authors declare that the research was conducted in the absence of any commercial or financial relationships that could be construed as a potential conflict of interest.
